# Effects of papain-based gel and air-abrasion enamel pretreatments on microleakage, sealant penetration, and shear bond strength of fissure sealant materials: an *in vitro* study

**DOI:** 10.3389/fdmed.2026.1908853

**Published:** 2026-08-21

**Authors:** Lozan Rashid, Aras Rauf

**Affiliations:** Department of Pedodontics and Community Oral Health, College of Dentistry, University of Sulaimani, Sulaymaniyah, Iraq

**Keywords:** air abrasion, fissure morphology, microleakage, papain-based gel, pit and fissure sealants, sealant penetration, shear bond strength

## Abstract

**Introduction:**

Establishing a durable seal between enamel and fissure sealant materials is critical for the long-term prevention of occlusal caries, particularly in anatomically complex pits and fissures. This *in vitro* study evaluated the effects of chemical pretreatment with a papain-based gel and mechanical pretreatment with air abrasion on the microleakage, sealant penetration, and shear bond strength of two pit and fissure sealant materials.

**Methods:**

A total of 120 sound human premolars were randomly allocated according to sealant type, resin-based sealant (RBS) or resin-modified glass ionomer cement (RMGIC), and pretreatment protocol: no pretreatment, enzymatic papain-based gel, or air abrasion. Following sealant application and thermocycling, microleakage was evaluated by dye penetration under a stereomicroscope. Sealant penetration was measured in relation to fissure morphology using ImageJ software, and shear bond strength was evaluated using a universal testing machine.

**Results:**

RBS showed significantly lower microleakage than RMGIC, indicating better sealing ability. Papain-based gel pretreatment significantly reduced microleakage in the RBS group without affecting sealant penetration or shear bond strength; however, no comparable improvement was observed in the RMGIC group. In contrast, air abrasion pretreatment did not produce a significant improvement in any of the evaluated outcomes. Fissure morphology significantly influenced sealant penetration, with wider fissure patterns demonstrating greater penetration.

**Discussion:**

These findings indicate that fissure sealant performance is primarily influenced by material properties and fissure morphology, while the benefit of enamel pretreatment appears to be material-dependent.

## 1 Introduction

Pits and fissures are protected sites for the accumulation of plaque and food debris and are therefore highly susceptible to dental caries (1). This susceptibility is particularly pronounced in newly erupted first permanent molars (2), where the process of enamel maturation is not yet complete and the occlusal morphology is frequently characterized by deep, narrow, and retentive fissures (3). The limited access to the fissures and the complex internal anatomy of these structures present a challenge for mechanical cleansing (4) and may also compromise the effectiveness of traditional preventive approaches, such as plaque control and topical fluoride application. Thus, pit and fissure sealants are commonly used as a preventive method to provide a protective barrier on vulnerable occlusal surfaces and reduce the risk of caries development (5). This method involves the application of a sealing material to susceptible pits and fissures, forming a mechanical barrier that restricts bacterial colonization and prevents access to fermentable substrates. There are many different sealant materials available for use in pediatric dental practice. Despite this, resin-based sealants (RBS) remain widely used because of their favorable retention and sealing ability. Glass ionomer sealants (GIS), including resin-modified glass ionomer cement (RMGIC), have the added advantage of fluoride release. Resin modification improves the handling and mechanical characteristics of the material. Compomer sealants are also available and combine some of the properties of resin composites and glass ionomer systems (6).

The efficacy of pit-and-fissure sealants is primarily dependent on their adhesion to enamel, long-term retention, and resistance to marginal microleakage (7). Acid etching plays an important role in this process because it creates microporosities in the enamel, increases surface energy, and allows the sealant material to penetrate the etched surface (8). However, acquired pellicle proteins and organic residues may interfere with the uniformity of the etching pattern and compromise bonding. Therefore, the removal or alteration of this organic layer before acid etching may enhance the interaction between enamel and resin-based materials (9). Numerous chemical and mechanical surface-preparation techniques have been investigated to reduce microleakage and improve retention. Chemical methods include sodium hypochlorite (NaOCl), silver diamine fluoride, and chemomechanical caries removal (CMCR) agents used for enamel deproteinization (10, 11). In contrast, mechanical techniques include bristle brush cleaning, enameloplasty, air abrasion, and Er:YAG laser treatment (7, 12).

Since 1975, CMCR agents have been used to improve patient tolerance and comfort during caries removal procedures (13). These agents selectively remove infected dentin while preserving the underlying affected dentin (14). CMCR agents are categorized according to their composition as either NaOCl-based or enzyme-based formulations. Papain-based gels are enzyme-based, NaOCl-free agents that act by breaking down partially degraded collagen within necrotic tissue. In addition to their traditional use in caries removal, papain-based gels have been investigated as enamel-deproteinizing agents (15). Previous studies have reported that their application before fissure sealant placement may reduce microleakage and improve sealant penetration compared with acid etching alone or other deproteinizing protocols (16). Air abrasion is a technique that facilitates the thorough cleaning of fissures before sealant placement. Its use has increased with the development of minimally invasive dentistry (17). This method primarily involves the propulsion of aluminum oxide (Al₂O₃) particles by compressed air (18). In addition, air abrasion mechanically roughens the enamel surface and removes debris from the fissures before sealant placement, potentially improving sealant adaptation and penetration by reducing unfilled spaces (19).

Chemical and mechanical enamel pretreatments can modify the enamel surface and enhance micromechanical retention, thereby improving the adaptation and retention of fissure sealants. However, direct comparisons between these pretreatment approaches remain limited, particularly when different fissure sealant materials are evaluated. Moreover, their effects on multiple key performance outcomes, including microleakage, sealant penetration, shear bond strength (SBS), and penetration in relation to fissure morphology, have not been fully investigated. This *in vitro* study aimed to assess and compare the effects of a papain-based gel and air abrasion as enamel pretreatment methods on the sealing and bonding performance of RBS and RMGIC. The outcomes were evaluated through microleakage analysis, quantitative measurement of sealant penetration in relation to fissure morphology, and SBS testing. The null hypothesis was that neither papain-based gel nor air abrasion used as an enamel pretreatment would significantly affect the microleakage, sealant penetration, or SBS of the tested fissure sealant materials.

## 2 Materials and methods

### 2.1 Study design, and sample selection

**Figure 1:**
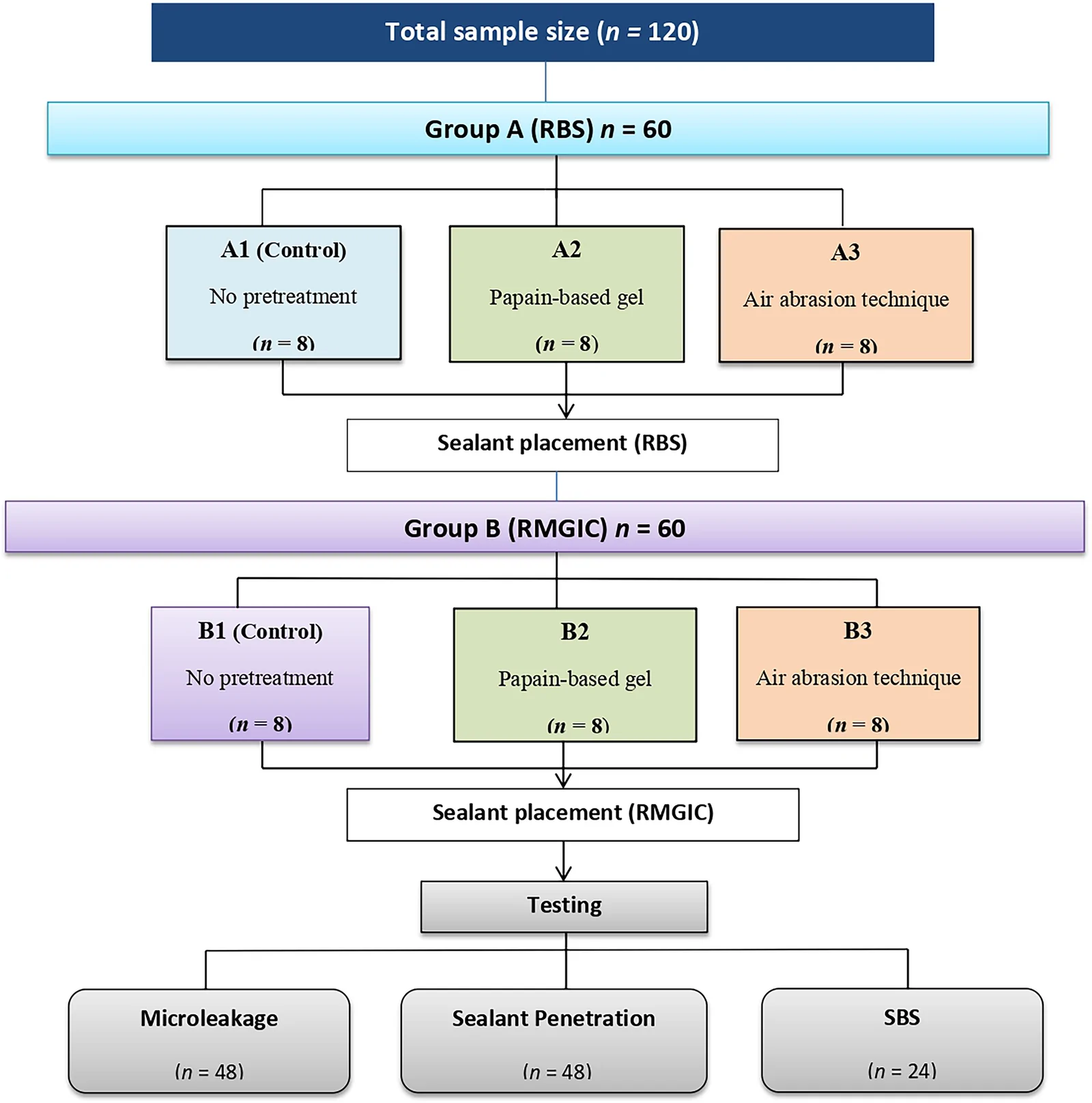
Study design flowchart.

This *in vitro* study was conducted in the Department of Pedodontics and Community Oral Health. Ethical approval was obtained from the Research Ethics Committee of the College of Dentistry, University of Sulaimani (Approval No. COD-EC-26-0137; 25 January 2026). The study evaluated the performance of two fissure sealant materials following enamel pretreatment with a papain-based gel or air abrasion. The assessed outcomes were microleakage, sealant penetration, and SBS. The configuration of the experimental groups is shown in Figure 1. An *a priori* sample size calculation was performed using G*Power software, version 3.1. The calculation was based on the *F*-test family using the ANOVA option (fixed effects, special, main effects, and interactions). An effect size of Cohen's *f* = 0.70 was derived from the SBS data reported in a previous study (10). With a significance level of *α* = 0.05, a statistical power of 80% (1−*β* = 0.80), six groups, and numerator degrees of freedom of 2, the required total sample size was 24 samples, (four per group). However, eight samples per group were ultimately included to enhance statistical reliability.

A total of 120 sound human premolars extracted for orthodontic reasons were collected from various orthodontic clinics and allocated to microleakage (*n* = 48), sealant penetration (*n* = 48), and SBS testing (*n* = 24). The collection and use of the extracted teeth were conducted in accordance with the ethical principles of the World Medical Association Declaration of Helsinki. Before tooth collection, each patient provided written informed consent authorizing the use of the extracted teeth for research purposes. Following collection, the teeth were debrided using a periodontal curette, disinfected in 0.5% chloramine-T solution for one week, and subsequently rinsed with distilled water. The occlusal surfaces were cleaned using a prophylactic brush mounted on a low-speed handpiece and then rinsed with water for 10 s. Each tooth was examined under a stereomicroscope to confirm its structural integrity. Teeth exhibiting demineralization, cracks, fractures, or cavitated carious lesions were excluded from the study.

### 2.2 Grouping procedure

The selected teeth were randomly allocated to six experimental groups using Microsoft Excel (Microsoft Corporation, Redmond, WA, USA). Each tooth was assigned a unique identification number, and the RAND() function was used to generate a random value for each tooth (20). The teeth were then sorted in ascending order according to the generated values and allocated to groups A1, A2, and A3 for RBS and groups B1, B2, and B3 for RMGIC.

RBS group:

Subgroup A1 (control): No pretreatment was performed. The teeth were etched with 37% phosphoric acid gel (Super Etch; SDI, Bayswater, VIC, Australia) for 15 s, rinsed for 30 s, and dried with oil- and water-free air. The sealant (Clinpro™, 3M ESPE, St. Paul, MN, USA) was applied using a syringe delivery device. The fissures were carefully filled with sealant, and a periodontal probe with a 0.5 mm tip diameter was used to eliminate air bubbles and ensure uniform distribution. To initiate the light-curing process, the occlusal surfaces were subjected to a light-curing apparatus (20A, Foshan Snyder Medical Equipment Co., Ltd., Foshan, China) in power mode (> 3000 mW/cm^2^) for 20 s, maintaining an average distance of 1–2 mm between the device and the sealant (21). Subgroup A2: The occlusal surfaces were pretreated with a papain-based chemomechanical caries removal gel (BRIX3000®, Brix Medical Science, Carcarañá, Argentina), which was applied with a microbrush applicator for 2 min according to the manufacturer's instructions. The gel was then extracted using an excavator, followed by comprehensive cleaning with water and air-drying. Etching and sealant application were then performed as described for subgroup A1 (16). Subgroup A3: Enamel surfaces were pretreated using an air abrasion device (COXO Air Abrasion Unit; COXO, China) with 50 μm Al₂O₃ particles applied uniformly over the occlusal surface at 4 bar pressure, 2 g/min powder flow rate, and 80 mL/min water flow rate, with the device positioned 2–3 mm from the surface at an angle of 45°–70°. The surfaces were then rinsed and dried before etching and sealant application as previously described (19).

RMGIC group:

Subgroup B1 (control): No additional enamel pretreatment was performed. The enamel surfaces were etched with 37% phosphoric acid gel for 5–10 s, rinsed with water for 30 s, and dried. The RMGIC (Riva Light Cure™; SDI, Victoria, Australia) capsules were mixed in a capsule mixer for 10 s according to the manufacturer's instructions. The material was then applied directly to the fissures using a disposable fine brush, allowed to flow for 10 s, and light-cured for 20 s (22). In subgroups B2 and B3, enamel pretreatment was performed using the papain-based gel and air abrasion, respectively, according to the previously described protocols. Enamel etching and RMGIC application were subsequently performed as described for subgroup B1.

### 2.3 Sample preparation for microleakage test

Following the pretreatment and sealant application procedures, the samples were stored in distilled water at 37 °C for 24 h. All samples were then subjected to 1,000 thermocycles between 5 °C and 55 °C, with a dwell time of 15 s in each water bath and a transfer time of 10 s between baths. The number of thermocycles used exceeded the 500-cycle artificial aging regimen described in ISO/TR 11405 and was consistent with previous *in vitro* studies evaluating dental adhesive materials and pit-and-fissure sealants (23, 24). Thereafter, two coats of acid-resistant nail varnish were applied to the tooth surfaces, leaving an exposed area of approximately 2 mm around the tooth–sealant interface. The specimens were subsequently immersed in a 1% methylene blue solution for 24 h at room temperature. They were then rinsed, cleaned, and allowed to air-dry before being sectioned buccolingually using a water-cooled, low-speed diamond saw (23). The sections were examined at ×40 magnification using a stereomicroscope (LAB-20; Optika, Ponteranica, Italy) to assess microleakage. The scoring criteria used for the microleakage evaluation are presented in Table 1. The morphological characteristics of the enamel–sealant interface were qualitatively assessed by examining and imaging one randomly selected sample from each group using scanning electron microscopy (SEM).

**Table 1:** Criteria for assessing microleakage.

Score	Definition
0	An absence of dye penetration.
1	Dye penetration up to one-third of the tooth–sealant interface.
2	Dye penetration between one-third and two-thirds of the tooth–sealant interface.
3	Dye penetration involving more than two-thirds of the tooth–sealant interface depth.

### 2.4 Sealant penetration analysis

**Figure 2:**
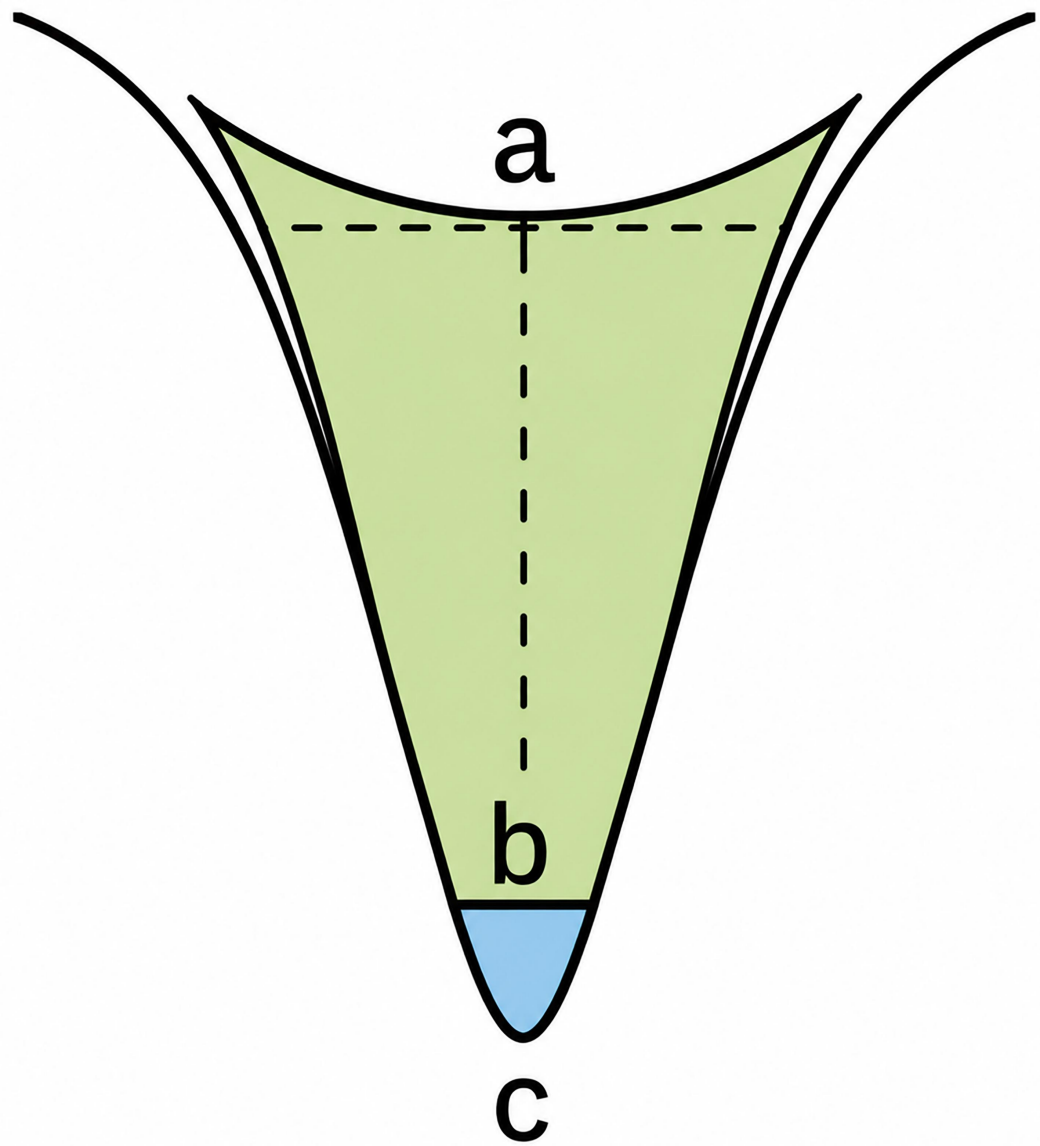
Schematic illustration of the measurement points used to calculate sealant penetration: **(a)** upper margin of the sealant, **(b)** base of the sealant, and **(c)** base of the fissure.

Sample preparation and sectioning were performed according to the protocol described for the microleakage assessment, but without dye immersion. Sealant penetration depth, total fissure length, unfilled space, and fissure morphology of each specimen were evaluated using a stereomicroscope at ×40 magnification. Fissure morphology was classified as V, I, U, or IK according to the observed fissure pattern. The following measurements were obtained, as illustrated in Figure 2 (25):

1. Sealant penetration depth: The vertical distance from the deepest point of the upper sealant margin (a) to the sealant base (b).
2. Length of Unfilled Space: Distance between the sealant base (b) and the fissure base (c).
3. Total length of the fissure: Distance from the deepest point of the upper sealant margin (a) to the base of the fissure (c).
4. Sealant penetration percentage: Calculated using the following formula:
  $$\begin{array}{rl} \;Sealant\;penetration\;(\text{\%})\;= & \;(sealant\;penetration\;depth\;\\ & /\;total\;fissure\;length)\;\times \;100 \end{array}$$

**Figure 3:**
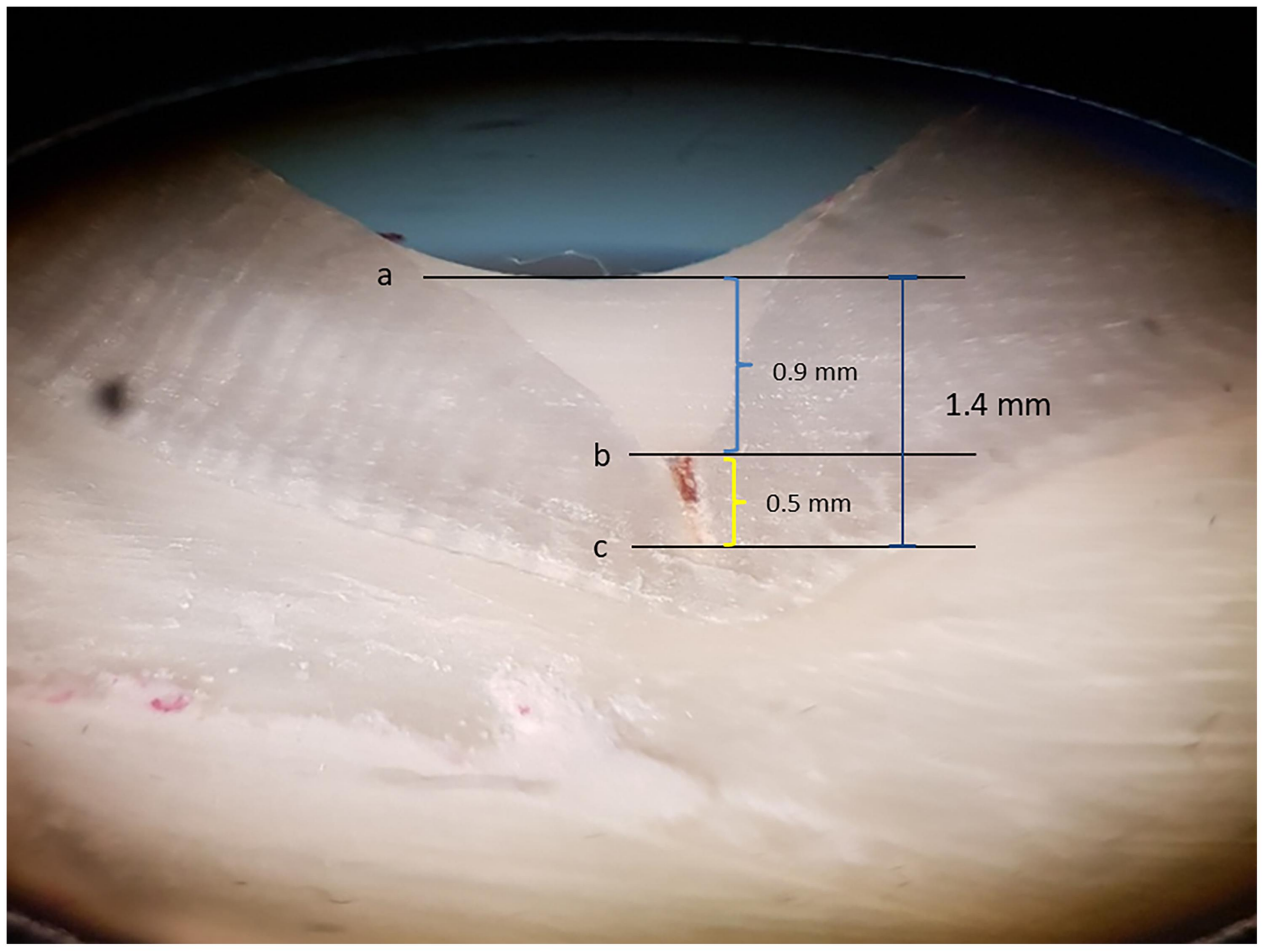
Microscopic view of a sectioned tooth showing the linear measurements used to calculate sealant penetration. The observed fissure morphology was I-type.

Linear measurements were made with ImageJ software (National Institutes of Health, Bethesda, MD, USA) and expressed in micrometers (µm). For each section, three repeated measurements were recorded, and the mean value was used for the final analysis, as shown in Figure 3. The data were systematically organized and subjected to statistical analysis.

### 2.5 Sample preparation for the SBS test

The crowns were embedded in self-curing acrylic resin (Pigeon Dental, China) within plastic rings, with the buccal surfaces facing outward. The midbuccal enamel surfaces were flattened using diamond fissure burs mounted on a high-speed handpiece and further polished with 320-grit silicon carbide abrasive paper under continuous water cooling. A cylindrical plastic mold measuring 3 mm in diameter and 3 mm in height was positioned perpendicular to the enamel surface, centered over the prepared area, and secured with adhesive wax. The sealant material was introduced into the mold and light-cured according to the protocol prescribed for each experimental group. After curing, the mold was carefully removed to obtain a standardized sealant cylinder. The specimens were stored in a humid environment at 37 °C for 24 h before testing. Each specimen was then individually secured in a custom jig attached to a universal testing machine (MultiTest 1-d; Mecmesin Ltd., West Sussex, UK). A knife-edged loading blade was positioned parallel to the enamel surface at the tooth–sealant interface, and shear force was applied until debonding occurred (26).

Shear bond strength (MPa) = F/A, where F is the failure load in newtons (N), and A is the bonded area in mm^2^.

Following debonding, the fractured surfaces were evaluated under a stereomicroscope at 40× magnification. Failure modes were classified as adhesive failure, characterized by the absence of sealant on the enamel surface; cohesive failure, defined by fractures occurring inside the sealant or enamel; and mixed failure, when both patterns were evident (26).

### 2.6 Blinding and intra-examiner reliability

All samples and their corresponding digital images were labeled with coded numerical identifiers, and the examiner remained blinded to group allocation during all outcome assessments. To evaluate intra-examiner reliability, microleakage scoring and the ImageJ-derived measurements used to calculate sealant penetration were repeated after a one-week interval without access to the initial results. Agreement between the two microleakage scoring sessions was assessed using weighted Cohen's kappa and interpreted according to the Landis and Koch criteria (27). The reliability of the ImageJ-derived sealant penetration percentages was evaluated using a two-way mixed-effects, absolute-agreement intraclass correlation coefficient (ICC) with a 95% confidence interval and interpreted according to the recommended ICC criteria (28). Almost perfect intra-examiner agreement was observed for microleakage scoring (weighted Cohen's *κ* = 0.948), while excellent intra-examiner reliability was observed for the ImageJ-derived sealant penetration percentages (ICC = 0.987; 95% CI: 0.977–0.993).

### 2.7 Statistical analysis

Statistical analyses were performed using IBM SPSS Statistics version 30. Microleakage scores were analyzed using the Kruskal–Wallis test followed, where appropriate, by Bonferroni-adjusted pairwise comparisons. Comparisons between the two sealant materials were performed using the Mann–Whitney *U* test. Sealant penetration and SBS were analyzed using two-way ANOVA. For both models, the normality of standardized residuals was assessed using the Shapiro–Wilk and Kolmogorov–Smirnov tests together with normal Q–Q plots, while homogeneity of variances was evaluated using Levene's test. Although slight deviations from normality were observed in the standardized residuals, homogeneity of variances was confirmed using Levene's test. Furthermore, the balanced experimental design with equal sample sizes across groups enhances the robustness of the two-way ANOVA to moderate violations of the normality assumption. The effect of fissure morphology on sealant penetration was evaluated using one-way ANOVA followed by Tukey's *post hoc* test. Failure-mode distributions were analyzed using Pearson's chi-square test with Monte Carlo exact significance. Statistical significance was set at *p* < 0.05.

## 3 Results

### 3.1 Microleakage evaluation

The distribution of microleakage scores and the corresponding subgroup comparisons within each sealant material are presented in Table 2. A statistically significant difference was detected among the RBS subgroups (*p* = 0.009). Bonferroni-adjusted pairwise comparisons showed that A2 (papain-based gel) exhibited significantly lower microleakage than A1 (control) (*p* = 0.011). Although A3 (air abrasion) demonstrated lower microleakage than A1, the difference did not reach statistical significance. No significant difference was observed between A2 and A3. In contrast, no significant difference was detected among the RMGIC subgroups (*p* = 0.836); therefore, *post hoc* pairwise comparisons were not performed.

**Table 2:** Microleakage scores and intersubgroup comparisons.

Groups	Score 0	Score 1	Score 2	Score 3	Median	Kruskal–Wallis comparison	Bonferroni-adjusted pairwise comparisons
RBS						*χ*^2^ (2) = 9.423 *p* = 0.009	
A1 (Control)	1	2	4	1	2		A1 vs. A2 *p* = 0.011*
A2 (Papain-based gel)	6	2	0	0	0		A1 vs. A3 *p* = 0.058
A3 (Air abrasion)	5	2	1	0	0		A2 vs. A3 *p* = 1.000
RMGIC						*χ*^2^ (2) = 0.359 *p* = 0.836	Not performed (overall Kruskal–Wallis test not significant)
B1 (Control)	0	0	3	5	3		
B2 (Papain-based gel)	0	0	3	5	3		
B3 (Air abrasion)	0	0	2	6	3		

Overall six-group comparison: Kruskal–Wallis *χ*^2^(5) = 34.086, *p* < 0.001*.

Overall RBS versus RMGIC comparison: Mann–Whitney *U* = 540.000, *Z* = 5.422, *p* < 0.001*.

Pairwise *p* values were Bonferroni-adjusted.

*Statistical significance was set at *p* < 0.05.

**Figure 4:**
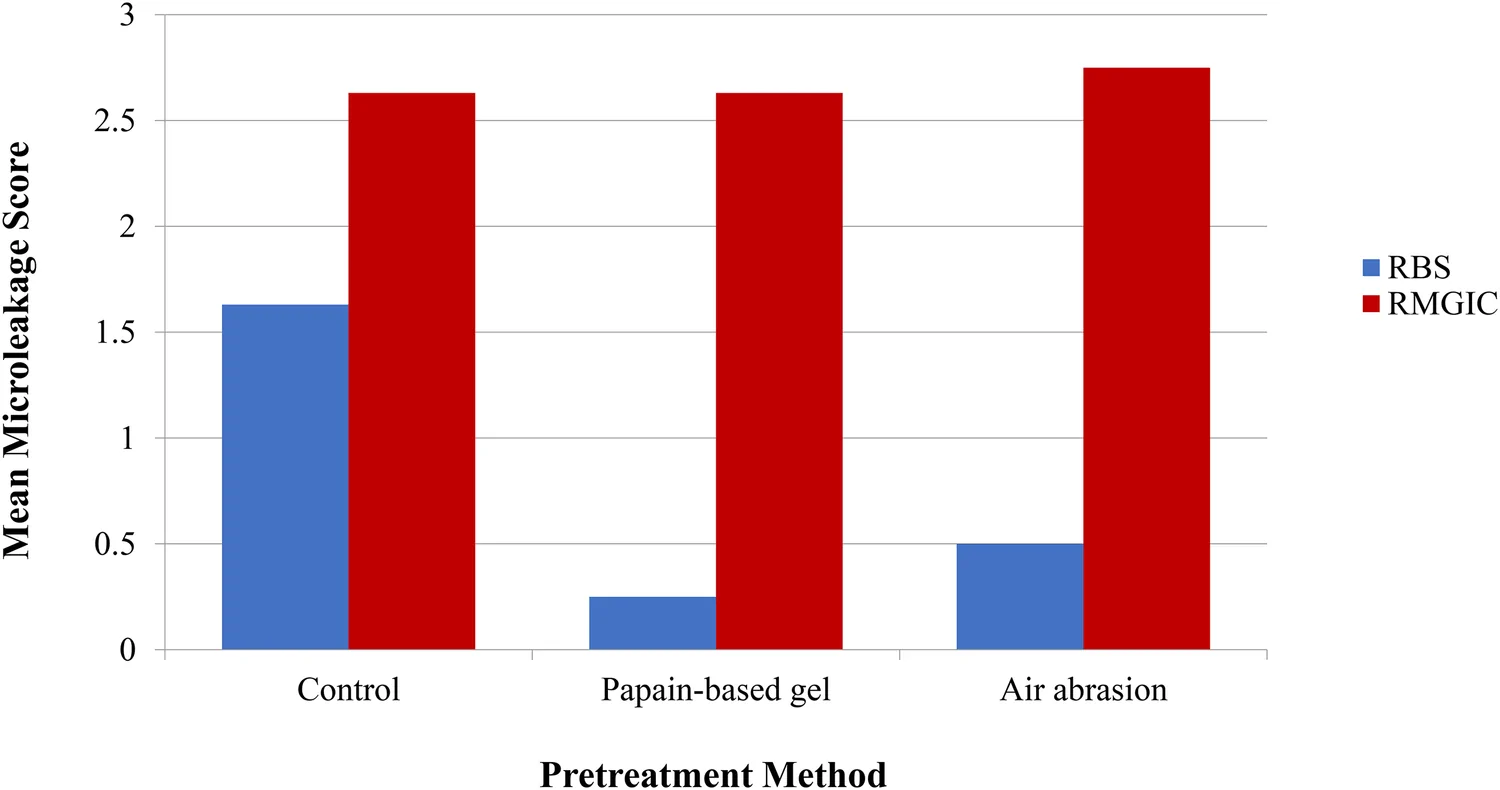
Mean microleakage scores of the RBS and RMGIC groups.

**Figure 5:**
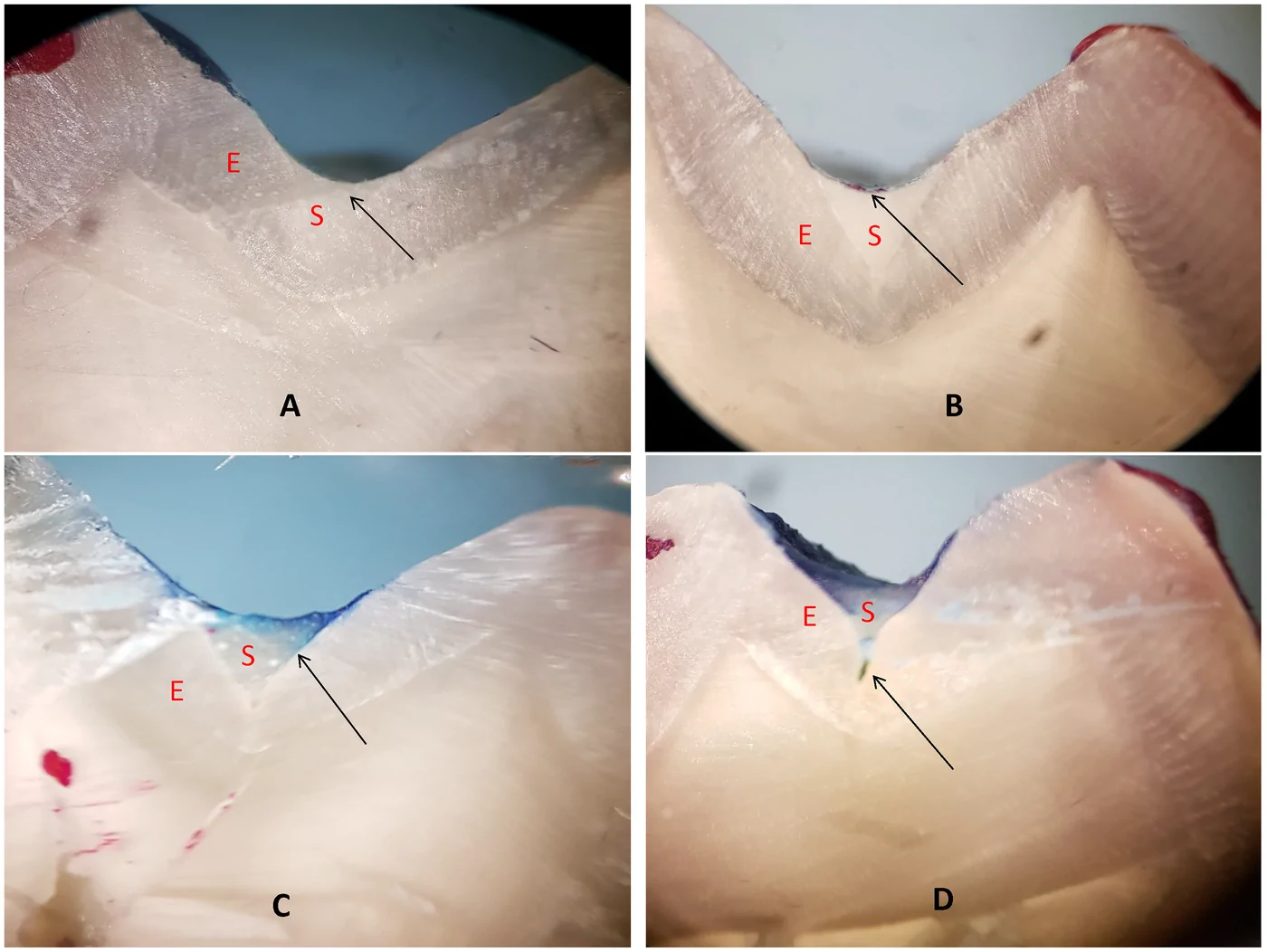
Microleakage scores between enamel (E) and sealant (S) under a light microscope. **(A)** Score 0, **(B)** Score 1, **(C)** Score 2, **(D)** Score 3. The enamel–sealant leakage area is indicated by black arrows.

**Figure 6:**
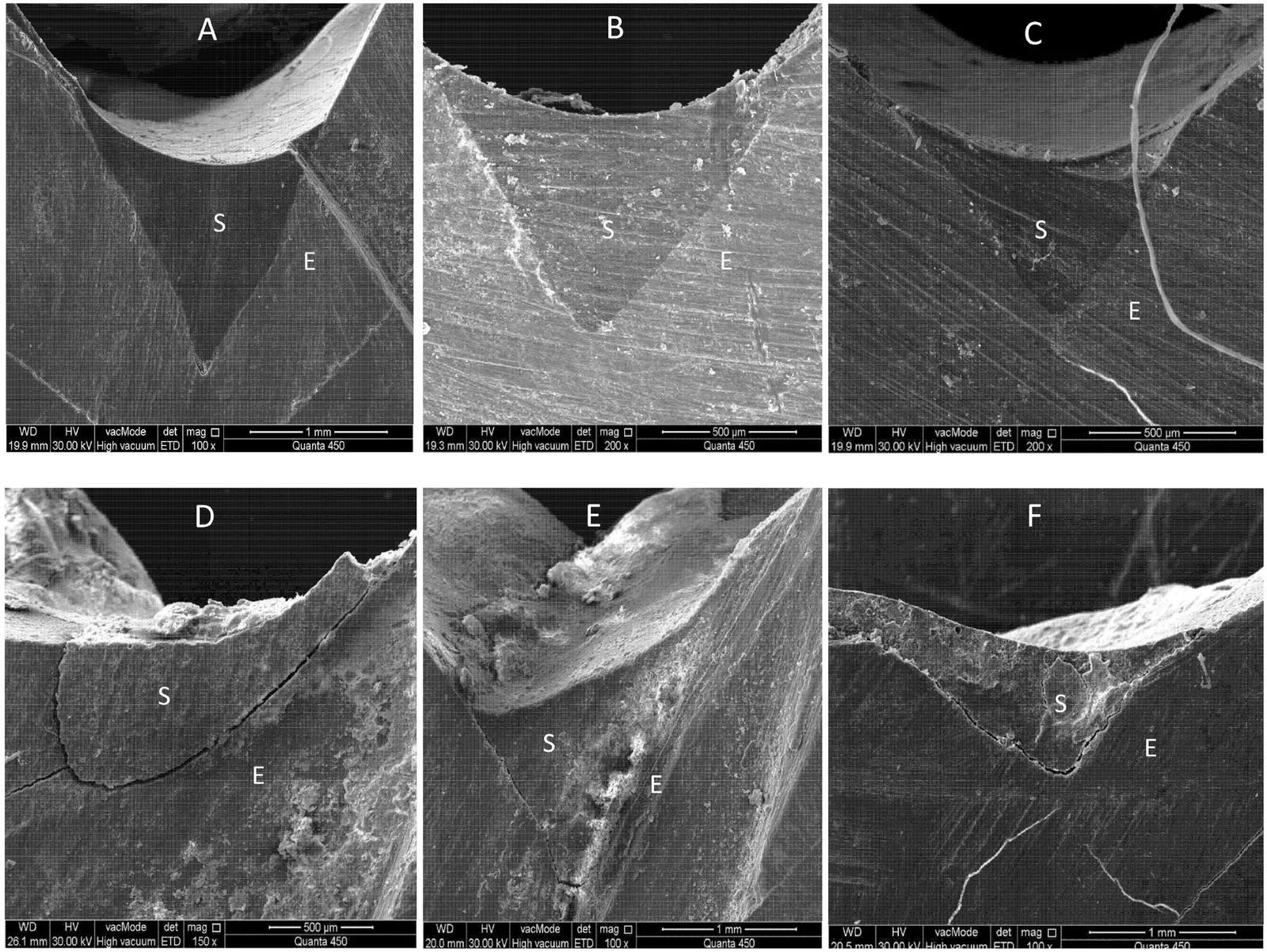
Representative scanning electron microscopy images of the enamel–sealant interface under different pretreatment conditions: **(A–C)** RBS groups and **(D–F)** RMGIC groups. **(A)** ×100, scale bar = 1 mm; **(B)** ×200, scale bar = 500 µm; **(C)** ×200, scale bar = 500 µm; **(D)** ×150, scale bar = 500 µm; **(E)** ×100, scale bar = 1 mm; **(F)** ×100, scale bar = 1 mm. E, enamel; S, sealant.

The overall comparison among the six study groups revealed a statistically significant difference in microleakage scores (*p* < 0.001). Furthermore, the RBS groups demonstrated significantly lower microleakage than the RMGIC groups, as illustrated in Figure 4. Representative stereomicroscopic microleakage patterns and SEM images are presented in Figures 5, 6, respectively. The qualitative SEM images appeared to show closer adaptation to the fissure walls and fewer visible interfacial gaps in the RBS specimens than in the RMGIC specimens.

### 3.2 Sealant penetration evaluation

The sealant penetration results and corresponding statistical analyses are presented in Table 3. Mean sealant penetration showed a descriptive increasing trend across the pretreatment protocols, from the control groups to the papain-based gel groups, with the highest mean values observed following air abrasion. However, neither sealant material nor pretreatment protocol had a statistically significant effect on sealant penetration, and no significant interaction was observed between these factors (all *p* > 0.05).

**Table 3:** Sealant penetration (%) according to sealant material, pretreatment method, and fissure morphology.

Variable	Category	Mean ± SD (%)	Test statistic	Effect size	*p*-value
Sealant material	RBS	84.00 ± 15.95	*F*(1,42) = 0.009	Partial *η*^2^ < 0.001	0.924
	RMGIC	84.45 ± 15.41			
Pretreatment method	Control	81.03 ± 16.71	*F*(2,42) = 0.769	Partial *η*^2^ = 0.035	0.470
	Papain-based gel	83.64 ± 15.97			
	Air abrasion	88.01 ± 13.92			
Interaction effect	Sealant material × pretreatment method	_	*F*(2,42) = 0.084	Partial *η*^2^ = 0.004	0.920
Fissure morphology	U-type	95.65 ± 4.11	*F*(3,44) = 20.317	*η*^2^ = 0.581 95% CI [0.348, 0.685]	<0.001*
	V-type	92.18 ± 6.74			
	I-type	77.17 ± 15.66			
	IK-type	60.27 ± 9.30			

Homogeneity of variance was satisfied (Levene's *F*(5, 42) = 0.236, *p* = 0.944).

Two-way ANOVA was used for sealant material, pretreatment method, and their interaction; one-way ANOVA followed by Tukey's *post hoc* test was used for fissure morphology.

*Statistical significance was set at *p* < 0.05; CI, confidence interval.

**Figure 7:**
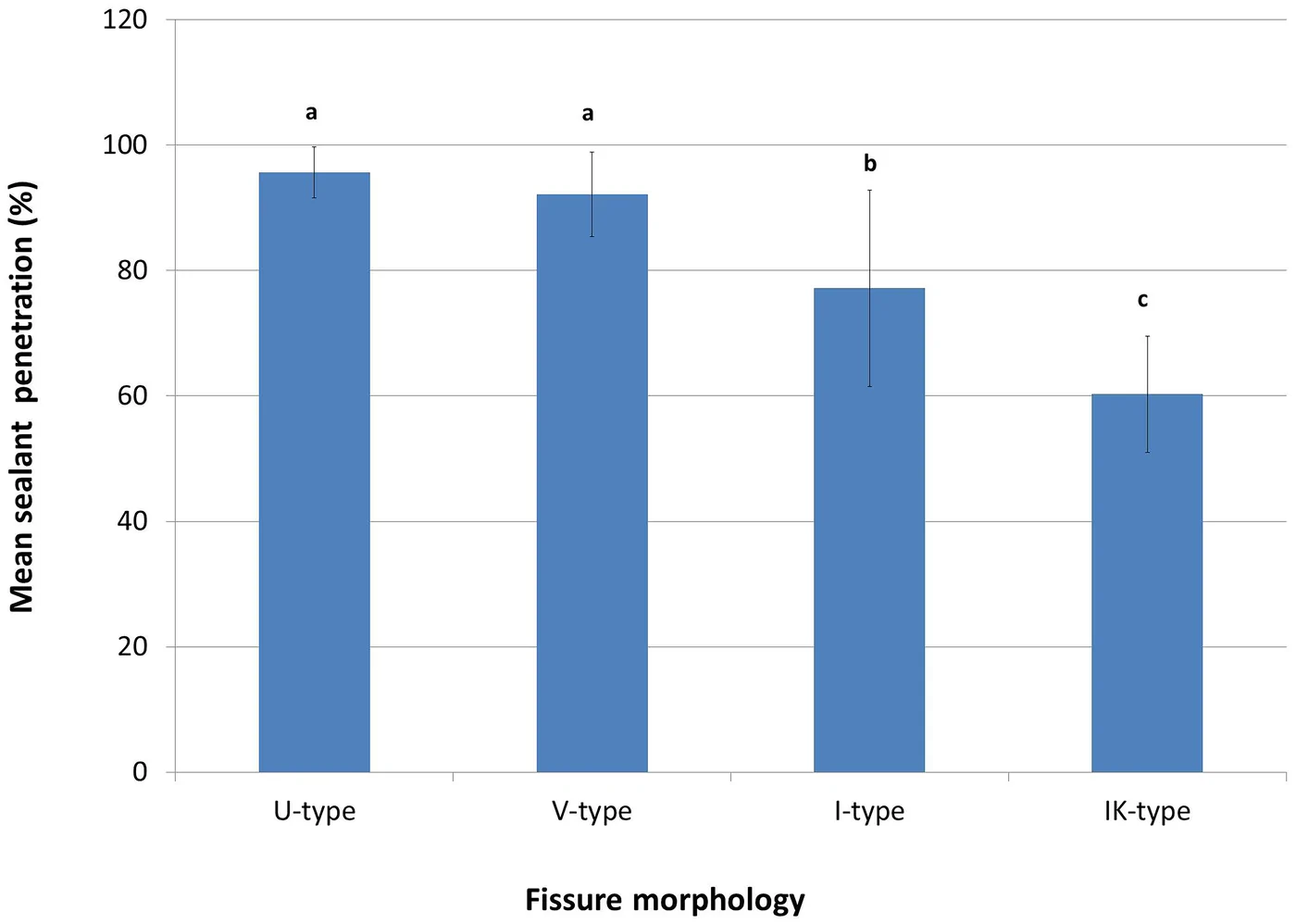
Mean sealant penetration percentage according to fissure morphology. Error bars indicate standard deviation. Different letters indicate significant differences (*p* < 0.05).

V-type fissures were the most frequently observed morphology (*n* = 17), followed by I-type (*n* = 15), U-type (*n* = 10), and IK-type fissures (*n* = 6). Fissure morphology had a statistically significant effect on sealant penetration (*p* < 0.001). The highest mean penetration percentages were observed in U-type (95.65%) and V-type fissures (92.18%), followed by I-type (77.17%) and IK-type fissures (60.27%). Tukey's *post hoc* analysis demonstrated significantly greater penetration in U- and V-type fissures than in I- and IK-type fissures. In addition, I-type fissures exhibited significantly greater penetration than IK-type fissures, whereas no statistically significant difference was observed between U- and V-type fissures, as illustrated in Figure 7.

### 3.3 Shear bond strength evaluation

**Figure 8:**
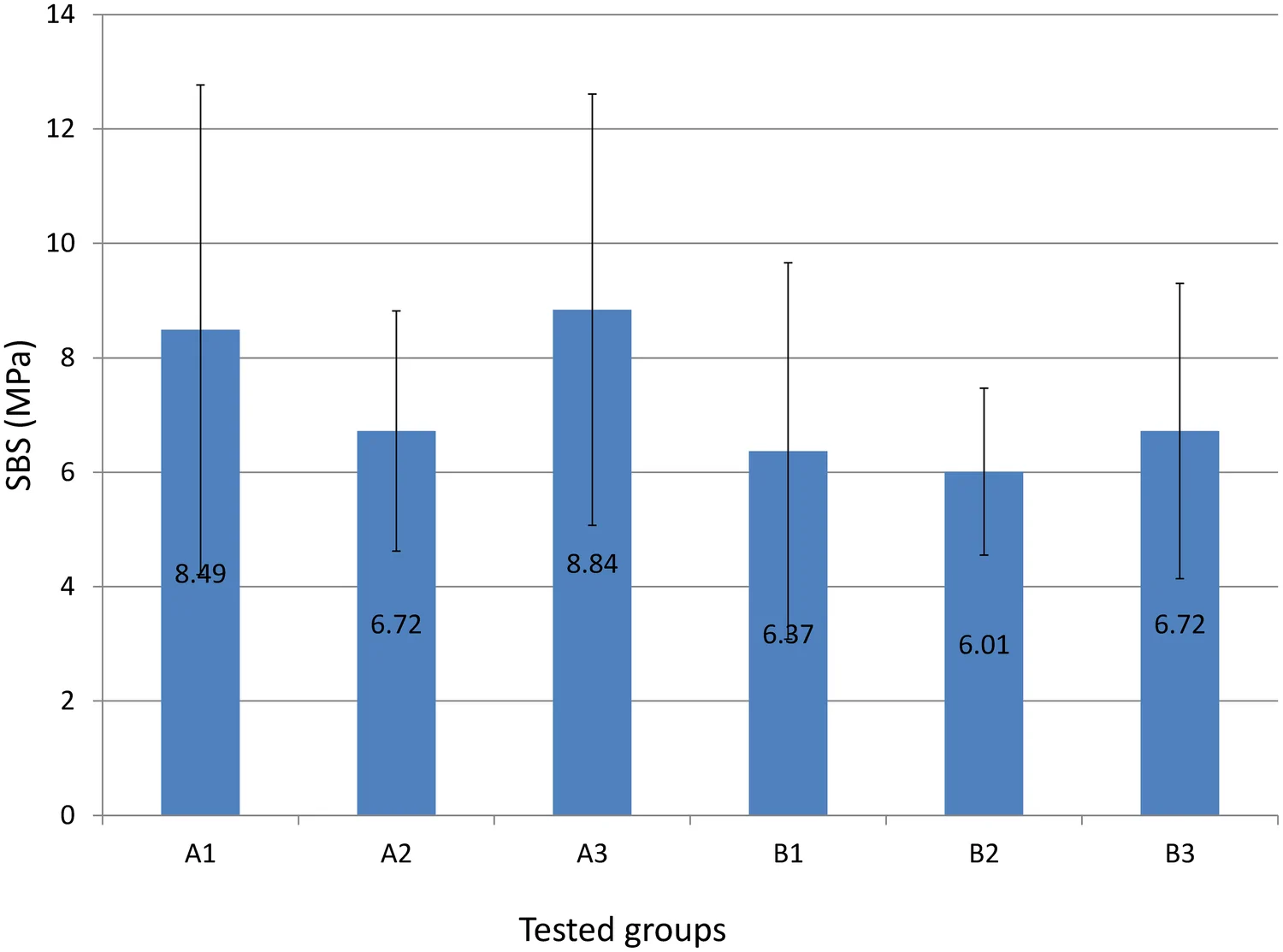
Mean shear bond strength values of the tested groups. Values inside bars represent means, and error bars indicate standard deviation.

**Figure 9:**
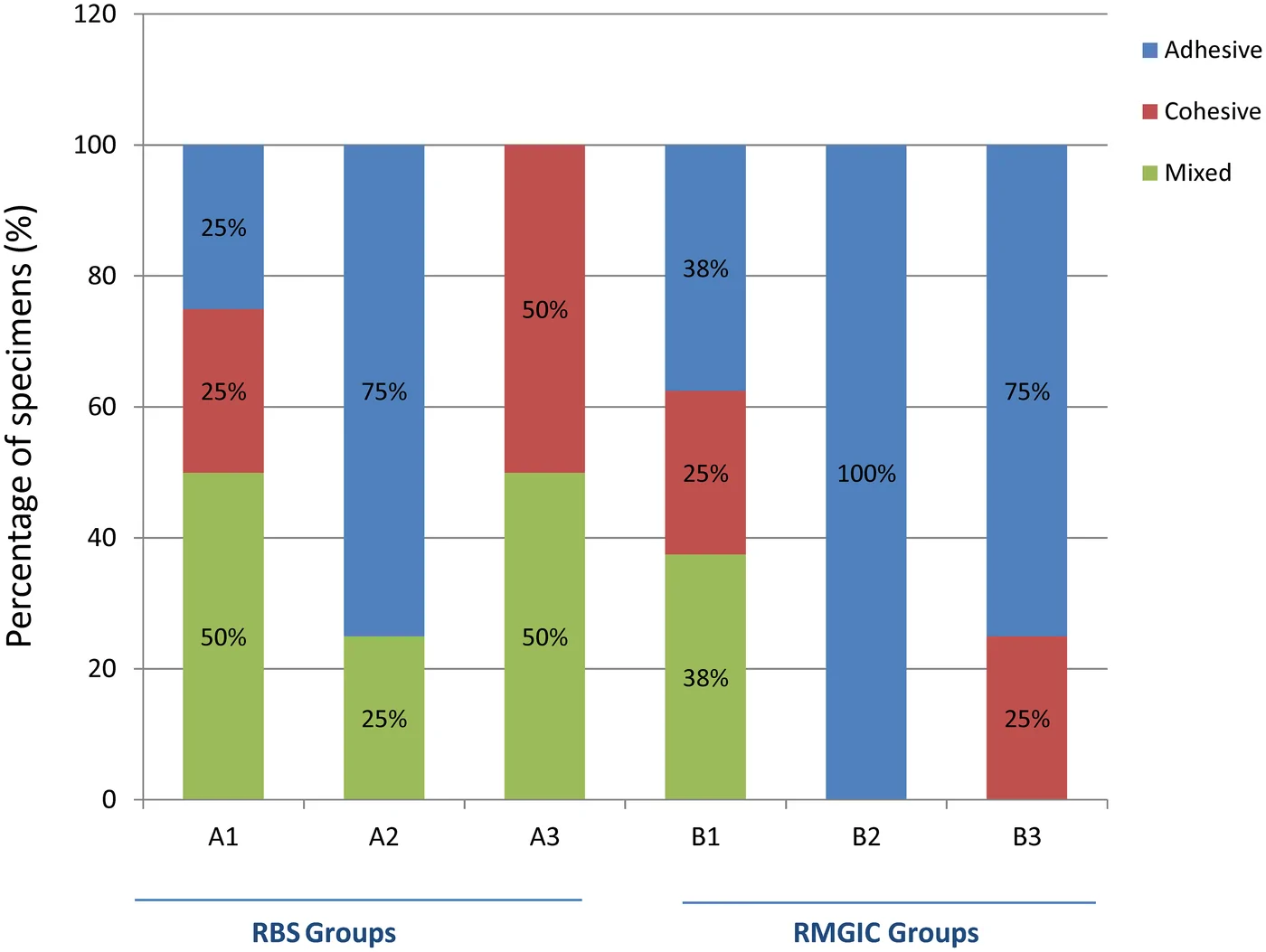
Proportional distribution of failure modes (adhesive, cohesive, and mixed) throughout the study groups.

The SBS results and corresponding statistical analyses are presented in Table 4. Although the RBS groups exhibited numerically higher mean values than the RMGIC groups, two-way ANOVA revealed no statistically significant main effect of sealant material on SBS. Similarly, the pretreatment protocol had no significant main effect, and no significant interaction was observed between sealant material and pretreatment protocol (all *p* > 0.05). The distribution of SBS values is illustrated in Figure 8. Failure-mode analysis revealed that adhesive failure was the most frequently observed mode (52.1%), followed by mixed failure (27.1%) and cohesive failure (20.8%). A statistically significant association was observed between the study groups and failure-mode distribution (Pearson's chi-square test, *p* = 0.005), and this association remained significant according to the Monte Carlo exact test (*p* = 0.004). Adhesive failures predominated in the RMGIC groups, whereas mixed and cohesive failures occurred more frequently in some RBS groups, as illustrated in Figure 9.

**Table 4:** Shear bond strength according to sealant material and pretreatment method.

Sealant material	Pretreatment method	Group	Mean ± SD (MPa)
Descriptive statistics			
RBS	Control	A1	8.49 ± 4.28
	Papain-based gel	A2	6.72 ± 2.11
	Air abrasion	A3	8.84 ± 3.77
RMGIC	Control	B1	6.37 ± 3.30
	Papain-based gel	B2	6.02 ± 1.47
	Air abrasion	B3	6.72 ± 2.59

Effect	Test statistic	Effect size	*p*-value
Two-way ANOVA results			
Sealant material	*F*(1, 42) = 3.465	Partial *η*^2^ = 0.076	0.070
Pretreatment method	*F*(2, 42) = 0.914	Partial *η*^2^ = 0.042	0.409
Sealant material × pretreatment method	*F*(2, 42) = 0.283	Partial *η*^2^ = 0.013	0.755

Homogeneity of variances was satisfied: Levene's *F*(5, 42) = 0.993, *p* = 0.434.

Two-way ANOVA was used to evaluate the main effects of sealant material and pretreatment method and their interaction.

Statistical significance was set at *p* < 0.05.

## 4 Discussion

The present *in vitro* study evaluated the effects of two enamel pretreatment protocols, namely chemical deproteinization using a papain-based gel and mechanical conditioning using air abrasion, on the performance of two commonly used pit and fissure sealant materials. The study design allowed standardized comparison of material and pretreatment variables that influence sealant performance. RBS are widely recognized for their superior micromechanical retention and enhanced wear resistance following conventional acid etching and application procedures. Acid etching selectively dissolves hydroxyapatite crystals to generate a microporous surface of enamel for the formation of resin tags and mechanical interlocking (29). In contrast, RMGIC combines the advantages of conventional glass ionomer cements with enhanced mechanical and handling characteristics due to resin incorporation (30). Despite the chemical adhesion of RMGIC, acid etching was performed in all study groups to standardize enamel conditioning and remove variability in surface preparation. The long-term clinical effectiveness of pit and fissure sealants depends on their retention on enamel and their ability to inhibit the accumulation of dental plaque (31). Several chemical pretreatment methods, including enamel deproteinization, have been proposed to modify the enamel surface. Papain-based gel may degrade organic material and improve enamel surface cleanliness, thereby promoting more favorable sealant–enamel interaction. They have also been described as biocompatible agents suitable for enamel pretreatment (10). Mechanical pretreatment techniques, such as air abrasion, have been introduced to modify the enamel surface morphology by removing debris and increasing surface roughness (19). Given the limited and conflicting evidence regarding the effectiveness of these pretreatment strategies, this study aimed to evaluate the influence of chemical and mechanical enamel pretreatment on the performance of pit-and-fissure sealants.

The present findings indicated that sealant material significantly influenced microleakage, with RBS exhibiting better sealing ability than RMGIC. This difference may be attributed to the distinct physicochemical properties and bonding mechanisms of the two materials. RBS materials have lower viscosity and better flow characteristics, which may facilitate deeper penetration into enamel microporosities and fissure irregularities. Moreover, they provide strong micromechanical retention through resin-tag formation and exhibit lower water sorption and solubility, potentially contributing to improved marginal adaptation and long-term sealing ability. In contrast, RMGIC materials are relatively more viscous and hydrophilic and may therefore be more susceptible to moisture-related effects, dissolution, and marginal degradation (32). Papain-based gel significantly reduced microleakage in the RBS group, possibly by improving the interaction between the RBS and acid-etched enamel and thereby enhancing marginal adaptation. No comparable effect was observed in the RMGIC group, suggesting that the benefit of this pretreatment may be material-dependent. Representative SEM images showed closer marginal adaptation and fewer visible interfacial gaps in the RBS specimens, supporting the stereomicroscopic findings. However, these images were interpreted as qualitative observations. The microleakage findings of the present study are consistent with those of Sridhar et al. (33), who reported significantly lower microleakage in RBS, and the long-term evidence by Petrauskiene et al. (29). However, available evidence suggests that enamel deproteinization does not consistently produce a significant reduction in microleakage, as demonstrated by Yamada et al. (34) and Bayrak et al. (35). Valencia et al. (36) reported that although pretreatment enhances enamel etching patterns and surface morphology, these improvements do not necessarily translate into reduced microleakage. Similarly, Ciucchi et al. (19) reported that air abrasion did not significantly reduce microleakage compared with conventional acid etching, whereas Bhadule et al. (37) found insufficient evidence that air-abrasion pretreatment improves the clinical retention of pit-and-fissure sealants. Collectively, these findings suggest that microleakage may be influenced more strongly by the intrinsic properties and bonding behavior of the sealant material than by additional enamel pretreatment. From a clinical perspective, papain-based gel adds a 2-min application step before acid etching, which may increase chairside time and complicate treatment in young, anxious, or poorly cooperative children (16). However, the significant reduction in microleakage observed in the RBS group suggests that this additional step may be beneficial in selected cases involving plaque-retentive or difficult-to-clean fissures. Air abrasion also requires specialized equipment, effective isolation, and additional procedural preparation; nevertheless, it may be advantageous for deep or anatomically complex fissures that are difficult to clean using conventional methods (37). Accordingly, both pretreatment methods should be considered selective adjuncts rather than routine procedures.

Regarding sealant penetration, neither sealant type nor pretreatment protocol produced a statistically significant effect. However, fissure morphology had a significant influence, with U- and V-shaped fissures showing greater penetration than I- and IK-shaped fissures, and the lowest values observed in the IK type. This finding agrees with Garg et al. (25), who identified fissure configuration as an important determinant of sealant penetration. The wider and more accessible anatomy of U- and V-shaped fissures likely facilitated sealant flow toward the fissure base, whereas the narrow openings and complex internal morphology of I- and IK-shaped fissures may have restricted penetration. The predominance of V-shaped fissures observed in the present study is consistent with Hristov et al. (38), who reported that this morphology is common in both primary and permanent teeth. The absence of a significant difference between the two sealant materials also agrees with Bishayi et al. (39), who found comparable penetration among different sealant types. Likewise, the lack of a significant pretreatment effect suggests that enamel surface modification may not overcome the anatomical restrictions of narrow and complex fissures. Collectively, these findings indicate that fissure morphology may influence sealant penetration more strongly than either sealant type or enamel pretreatment. Therefore, assessment of fissure anatomy may help clinicians anticipate potential limitations in sealant penetration during clinical application.

SBS was not significantly affected by either sealant type or pretreatment protocol, although the RBS groups exhibited slightly higher mean values than the RMGIC groups. No significant interaction was observed between the two factors, indicating that the effect of pretreatment on SBS was comparable for both materials. Under the conditions of the present study, therefore, additional chemical or mechanical enamel pretreatment did not improve bond strength. This finding is consistent with Ahmad et al. (40), who reported no statistically significant improvement in SBS following enamel pretreatment. Similarly, Bhushan et al. (41) found that additional air-abrasion pretreatment did not significantly improve the clinical retention of pit-and-fissure sealants. The absence of a significant difference between RBS and RMGIC also agrees with Waghmode et al. (42). Failure-mode analysis provided additional insight into the bonding behavior of the tested sealants. Adhesive failure predominated in the RMGIC groups, indicating that fracture occurred mainly at the enamel–sealant interface. This pattern may be related to the bonding mechanism of RMGIC, which relies partly on ionic interactions with the tooth structure and may provide less micromechanical interlocking. In contrast, mixed and cohesive failures occurred more frequently in some RBS groups, indicating that fracture was not confined exclusively to the bonded interface. This pattern may reflect stronger micromechanical interlocking between RBS and acid-etched enamel. Although SBS values did not differ significantly among the study groups, the significant variation in failure-mode distribution indicates that specimens with comparable bond strength values may fail through different mechanisms depending on the characteristics of the enamel–sealant interface (32, 43).

This study has several limitations that should be acknowledged. First, the relatively small sample size within each subgroup may have reduced the statistical power to detect subtle differences among the tested materials and pretreatment protocols. Second, although thermocycling was performed to simulate thermal changes in the oral cavity, the use of 1,000 thermocycles represents relatively short-term artificial aging. Moreover, the *in vitro* design cannot fully reproduce the complexity of the clinical environment, including salivary contamination, pH fluctuations, masticatory loading, biofilm formation, and long-term moisture exposure. Third, SEM analysis was performed on only one representative specimen from each experimental group; therefore, the SEM findings should be considered qualitative and illustrative rather than quantitative evidence of intergroup differences. Furthermore, enamel surface roughness, plaque accumulation, and color-stability were not evaluated. Future studies should incorporate larger sample sizes, extended artificial aging protocols, SEM evaluation of multiple specimens per group, surface roughness analysis, microbiological assessment, color stability evaluation, and well-designed clinical trials to validate the present findings under long-term intraoral conditions.

## 5 Conclusions

RBS demonstrated significantly better marginal sealing ability than RMGIC. Papain-based gel significantly reduced microleakage in RBS, whereas neither papain-based gel nor air abrasion significantly affected sealant penetration or SBS. Fissure morphology was the principal determinant of sealant penetration, with wider fissure configurations demonstrating greater penetration percentages than narrower and more complex fissure types. These results suggest that the clinical performance of fissure sealants is determined mainly by the intrinsic properties of the sealing material and the anatomical configuration of the fissure, rather than by routine enamel pretreatment alone. Therefore, careful selection of sealant material and thorough evaluation of fissure morphology may play a decisive role in improving the long-term clinical success of pit and fissure sealants.

## Data Availability

The raw data supporting the conclusions of this article will be made available by the authors, without undue reservation.
