# Supplementary material for: Effects of papain-based gel and air-abrasion enamel pretreatments on microleakage, sealant penetration, and shear bond strength of fissure sealant materials: an in vitro study
Source: Front Dent Med. 2026 Aug 21;7:1908853. doi: 10.3389/fdmed.2026.1908853 (PMC13541521; doi:10.3389/fdmed.2026.1908853)
Supplement: Supplementary file 1 [file Datasheet1.pdf]

Consent Form of Using Extracted Teeth in Research

College of Dentistry

Researcher: Dr. Lozan Mohammed Rashid
Supervisor: Prof. Dr. Aras Maruf Rauf

Patient name: ___________________________

Age: _______________
Gender: ☐ Male ☐ Female

Date: _______ / _______ / _______

I understand that my tooth/teeth will be extracted as part of my orthodontic treatment plan.

I have been informed that my extracted tooth/teeth will be collected and used in a laboratory dental research study.

I understand that donating my extracted tooth/teeth for research is completely voluntary. Refusing to donate my tooth/teeth will not affect my orthodontic treatment in any way.

I understand that no additional treatment, procedure, pain, or risk will be added because of this research.

I understand that my personal information will be kept confidential. My name or identity will not appear in any publication or report related to this study.

Patient signature: __________________ **Researcher signature:** ____________________

- **For patients under 18 years old**

I am the parent/legal guardian of the patient named above, and I give permission for my child's extracted tooth/teeth to be used in this research study.

Parent/Guardian name: _______________________________

Relationship to patient: ______________________________

Parent/Guardian signature: ____________________________

Date: _______ / _______ / _______

College of Dentistry

فۆرمی ڕەزامەندی بۆ بەکارهێنانی ددانی هەڵکێشراو لە توێژینەوەدا

توێژەر: د. لۆزان محمەد ڕە شید

سەرپەرشتیار: پ.د. ئاراس مەعروف ڕووف

ناوی نەخۆش: ____________________________

تەمەن: _______________ **ڕەگەز:** نێر ☐ مێ ☐

بەروار: ______ /_______ /_______

من تێگەیشتووم کە ددان/ددانەکانم وەک بەشێک لە پلانی چارەسەری ڕاستکردنەوەی ددان هەڵدەکێشرێن.

ئاگادارکراومەتەوە کە ددانە هەڵکێشراوەکانم بەکاردەهێنرێن بۆ مەبەستی توێژینەوەی زانستی.

من تێگەیشتووم کە بەخشینی ددانە هەڵکێشراوەکانم بۆ توێژینەوە بە تەواوی ئارەزوومەندانەیە. ڕەتکردنەوەی بەخشینی ددانەکان هیچ کاریگەرییەک لەسەر پروسەی ڕاستکردنەوەی ددانەکانم نابێت.

من تێگەیشتووم کە هیچ چارەسەر، کارێکی زیادە، ئازار، یان مەترسییەکی زیادە بەهۆی ئەم توێژینەوەیەوە بۆ من زیاد ناکرێت، چونکە ددانەکان تەنها دوای هەڵکێشان بەکاردەهێنرێن.

من تێگەیشتووم کە زانیارییە کەسییەکانم بە نهێنی دەمێننەوە. ناو یان ناسنامەم دەرناکەوێت لە هیچ بڵاوکراوە و ڕاپۆرتێکدا.

واژۆی نەخۆش: _____________________ واژۆی توێژەر: _________________________

بۆ نەخۆشانی خوار تەمەنی ١٨ ساڵ:

من دایک/باوک یان سەرپەرشتیاری یاسایی نەخۆشی ناوبراوم، و ڕەزامەندی دەدەم کە ددانە هەڵکێشراوەکانی منداڵەکەم لەم توێژینەوەیەدا بەکاربهێنرێن.

ناوی دایک/باوک یان سەرپەرشتیار: ________________________________

پەیوەندی بە نەخۆشەوە: _______________________

واژۆی دایک/باوک یان سەرپەرشتیار: ____________________

بەروار: ______ /_______ /_______
